# Prevalence of multimodal treatment in children and adolescents with ADHD in Germany: a nationwide study based on health insurance data

**DOI:** 10.1186/s13034-021-00431-0

**Published:** 2021-12-18

**Authors:** Oliver Riedel, Simon Klau, Ingo Langner, Christian Bachmann, Oliver Scholle

**Affiliations:** 1 Leibniz Institute for Prevention Research and Epidemiology – BIPS, Achterstrasse 30, 28359 Bremen, Germany; 2 Department of Child & Adolescent Psychiatry, University Hospital Ulm, Ulm University, Steinhövelstrasse 5, 89075 Ulm, Germany

**Keywords:** Attention-deficit hyperactivity disorder, ADHD, Treatment, Pharmacotherapy, Psychotherapy, Multimodal therapy, Health claims data

## Abstract

**Background:**

Attention-deficit hyperactivity disorder (ADHD) ranks top among neurodevelopmental disorders in children and adolescents. Due to a large number of unfavorable outcomes including psychiatric comorbidities, school problems, and lower socioeconomic status, early and effective treatment of ADHD is essential. Multimodal treatment has become the gold standard in ADHD management, comprising pharmacotherapy and psychosocial interventions, e.g., psychotherapy. Yet, little is known about the prevalence of multimodal treatment in routine care.

**Methods:**

Based on German health claims data for the years 2009–2017, we identified children and adolescents aged 3–17 years diagnosed with ADHD and characterized them cross-sectionally (per calendar year) in terms of treatment status and psychiatric comorbidities. The detection of pharmacotherapy was based on dispensations of drugs to treat ADHD (e.g., methylphenidate); psychotherapeutic treatment was based on corresponding billing codes. Multimodal treatment was assumed if ADHD medication and psychotherapeutic treatment were coded within the same calendar year. Psychiatric comorbidities were based on outpatient and inpatient diagnoses. Prevalences of ADHD and proportions of different treatment options were calculated and standardized by age and sex.

**Results:**

In 2017, 91,118 children met the study criteria for ADHD (prevalence: 42.8/1000). Of these, 25.2% had no psychiatric comorbidity, 28.8% had one, 21.6% had two, and 24.5% had three or more. Regarding overall treatment status, 36.2% were treated only pharmacologically, 6.5% received multimodal treatment, and 6.8% were treated with psychotherapy only (neither treatment: 50.2%). With increasing numbers of psychiatric comorbidities, the proportions of patients with multimodal treatment increased from 2.2% (no psychiatric comorbidities) to 11.1% (three or more psychiatric comorbidities) while the proportions of untreated (from 56.8% to 42.7%) or only pharmacologically treated patients (38.4% to 35.0%) decreased. From 2009 to 2017, prevalences were stable and the proportion of patients with only pharmacotherapy decreased from 48% to 36.5%. Concurrently, the proportion of patients with neither pharmacotherapy nor psychotherapy increased from 40.5% to 50.2%. The fraction of patients with multimodal treatment ranged between 6.5% (2017) and 7.4% (2013).

**Conclusions:**

Multimodal treatment, although recommended as the standard of treatment, is rather the exception than the rule. It is, however, increasingly common in ADHD patients with psychiatric comorbidities.

**Supplementary Information:**

The online version contains supplementary material available at 10.1186/s13034-021-00431-0.

## Background

Attention-deficit hyperactivity disorder (ADHD) ranks top among neurodevelopmental disorders in children and adolescents, with an estimated worldwide prevalence of 5.3% and a proportion of up to 65% of patients with ADHD symptoms persisting into adulthood [1, 2]. It is associated with unfavorable outcomes, including higher rates of accidents, delinquency, school problems, and consequently lower social status later in life [3–5]. Moreover, ADHD is also associated with a substantial risk of mental comorbidities, including depression or substance use disorders, which might affect up to 65% of all patients with ADHD [1, 6].

The current international gold standard in ADHD management in children and adolescents is a multimodal treatment approach, i.e., a combination of psychosocial interventions (e.g., psychotherapy) and pharmacotherapy with stimulants [7–9]. While the current German ADHD guidelines do not specifically suggest long-term combination therapies, they do suggest combination treatment for certain patient groups and/or clinical conditions (mainly residual ADHD symptoms). However, these suggestions do not specify the recommended duration of treatment. The guidelines also state that there is a need for longitudinal studies on a number of subjects, including the long-term effectiveness of combination therapies, which may explain the rather non-specific recommendation of combination therapy. In Germany, there are generally two ways of providing psychotherapeutic interventions. First, guideline-based psychotherapy comprises recognized therapies such as cognitive behavioral therapy and is limited to psychotherapists or licensed physicians of other specialties (e.g., psychiatrists). Moreover, it requires an extensive application for reimbursement by health insurance providers, including a comprehensive written statement on the expected success of treatment before starting therapy. The second treatment option comprises non-guideline-based psychotherapeutic interventions or counseling (“other non-drug psychiatric/psychotherapeutic treatment”). Unlike guideline-based psychotherapy, these interventions do not necessarily require psychotherapeutic approbation and are more easily reimbursed by health insurances providers. Thus, despite lower fees, the so-called other non-drug psychiatric/psychotherapeutic treatment could be an important additional pillar in the psychotherapeutic care of patients with ADHD. Importantly, both ways of providing psychotherapeutic interventions—guideline-based psychotherapy and other non-drug psychiatric/psychotherapeutic treatment—are fully reimbursed by health insurance providers in Germany, facilitating access to this type of care for the patients. Nonetheless, a recently published longitudinal analysis [10] revealed that less than one third of children with incident ADHD are treated with guideline-based psychotherapy in addition to pharmacotherapy. Cross-sectional analyses of the care situation across several calendar years are lacking. Moreover, it remains unclear, whether the sole consideration of guideline-based psychotherapy will sufficiently describe psychotherapeutic care of children with ADHD. However, there are hardly any data available on the prevalence of multimodal treatment of ADHD patients in routine care. Regarding patient-centered psychotherapeutic treatment, it is unknown whether additional consideration of low-threshold services such as other non-drug psychiatric/psychotherapeutic treatment would increase the proportion of patients with ADHD identified as recipients of psychotherapy.

The present study therefore aimed at characterizing the use of (non-)multimodal treatment among children and adolescents (age 3–17) diagnosed with ADHD in routine care in Germany between 2009 and 2017, considering: (a) the proportion of children and adolescents with ADHD receiving (non-)multimodal treatment (including trend analyses), (b) the association of (non-)multimodal treatment and psychiatric comorbidities, and (c) the increase of the proportion of patients with multimodal treatment if other non-drug psychiatric/psychotherapeutic treatment are also considered.

## Methods

### Data source

Data source for this study was the German Pharmacoepidemiological Research Database (GePaRD) which comprises claims data from four statutory health insurance (SHI) providers in Germany. It currently includes information on approximately 25 million persons who have been insured with one of the participating providers since 2004 or later [11]. GePaRD comprises demographic information, data on drug dispensations including the anatomical-therapeutic-chemical (ATC) code and outpatient services, which are coded according to the German Uniform Reimbursement Catalogue (*Einheitlicher Bewertungsmaßstab* = EBM catalogue). Diagnoses are coded according to the *International Statistical Classification of Diseases and Related Health Problems 10th Revision, German Modification* (ICD-10-GM). Per data year, there is information on approximately 20% of the general population available and all geographical regions of Germany are represented.

### Study design and study population

Data were obtained from annual cross-sectional studies comprising the calendar years 2009–2017. Detailed characteristics of the study population and (non-)multimodal treatment were shown for the most recent calendar year (i.e., 2017). Time trend analyses were based on all calendar years.

To be eligible for the study population, subjects had to meet the following inclusion criteria: (a) valid information on age and sex available, (b) being insured for at least 1 day in each quarter of the respective year, and (c) being 3–17 years of age. Accordingly, missing these criteria resulted in exclusion from the potentially eligible study population. Further exclusion criteria were not defined in order to represent the routine care in a population that is as unselected as possible. To be considered as diagnosed with ADHD, subjects had to meet a case definition, which has been described in detail before [10]. In brief, subjects were required to have either one inpatient diagnosis of ADHD (ICD-10-GM F90 or F98.8), at least two outpatient diagnoses of ADHD, or an outpatient diagnosis of ADHD and a dispensation of an ADHD drug (see “Assessment of treatment status” section below).

### Assessment of treatment status

For each calendar year, it was assessed whether patients diagnosed with ADHD were treated with medication and/or psychotherapeutic interventions. Pharmacotherapy of ADHD was assumed in case of at least one dispensation of methylphenidate (ATC-code: N06BA04), atomoxetine (N06BA09), dexamfetamine (N06BA02), lisdexamfetamine (N06BA12) or guanfacine (N06BA21), i.e., all drugs approved to treat ADHD during the study period in Germany.

The assessment of psychotherapeutic interventions was based on billing codes from the EBM catalogue and was determined by existence of interventions related to guideline-based psychotherapy and/or other non-drug psychiatric/psychotherapeutic treatment. An individual was assumed to have received psychotherapy if either at least one treatment from the guideline-based psychotherapy (including depth psychotherapy, analytical psychotherapy, and cognitive behavioral therapy, see Additional file 1: Table S1 for the corresponding EBM billing codes) or interventions from other non-drug psychiatric/psychotherapeutic treatment (see Additional file 2: Table S2 for the corresponding EBM billing codes) were billed for that person. Following recommendations by Herpertz et al. [12], psychotherapeutic interventions were classified as other non-drug psychiatric/psychotherapeutic treatment if they met minimum billing requirements in terms of their number (at least six interventions), density (at least six interventions within 6 months), and duration (at least 20 min in 1 day).

Multimodal treatment was assumed if a subject received pharmacotherapy and psychotherapeutic interventions in the respective calendar year. Accordingly, non-multimodal treatment was assumed if subjects received only one of these treatment options in the respective calendar year.

### Assessment of psychiatric comorbidities

Psychiatric comorbidities were assumed to be present if at least one corresponding inpatient or outpatient diagnosis (ICD-10-GM codes see Table 1) was recorded in the respective calendar year.

**Table 1:** Psychiatric comorbidities of children/adolescents with ADHD in 2017

	Sex		Total (n = 91,118)
	Male (n = 67,194)	Female (n = 23,924)	
Psychiatric comorbidities (ICD-10 codes), n (%)			
Depressive disorders (F32, F33, F41.2, F43.2)	7844 (11.7)	3807 (15.9)	11,651 (12.8)
Reaction to severe stress and adjustment disorders (F43.0, F43.1, F43.8, F43.9)	1906 (2.8)	1017 (4.3)	2923 (3.2)
Mental retardation (F70-F79)	1739 (2.6)	758 (3.2)	2497 (2.7)
Tic disorders (F95)	2567 (3.8)	463 (1.9)	3030 (3.3)
Specific developmental disorders of speech and language (F80)	14,984 (22.3)	4949 (20.7)	19,933 (21.9)
Specific developmental disorders of scholastic skills (F81)	12,685 (18.9)	5225 (21.8)	17,910 (19.7)
Specific developmental disorder of motor function (F82)	11,121 (16.6)	2884 (12.1)	14,005 (15.4)
Mixed specific developmental disorders (F83)	6813 (10.1)	2100 (8.8)	8913 (9.8)
Pervasive developmental disorders (F84.0, F84.1, F84.5, F84.8, F84.9)	4266 (6.3)	841 (3.5)	5107 (5.6)
Conduct disorders (F90.1, F91, F92)	20,871 (31.1)	4933 (20.6)	25,804 (28.3)
Phobic anxiety disorders (F40, F41.0, F41.1, F41.3, F41.8, F41.9, F93)	11,946 (17.8)	5248 (21.9)	17,194 (18.9)
Disorders of social functioning with onset specific to childhood and adolescence (F94)	2105 (3.1)	873 (3.6)	2978 (3.3)
Substance use disorders (F10-F19)	765 (1.1)	286 (1.2)	1051 (1.2)
Eating disorders (F50.0, F50.1, F50.2, F50.3, F50.4, F50.8, F50.9)	550 (0.8)	480 (2.0)	1030 (1.1)
Psychotic disorders (F20-F22, F25)	60 (0.1)	23 (0.1)	83 (0.1)
Obsessive–compulsive disorder (F42)	553 (0.8)	258 (1.1)	811 (0.9)
Dissociative/conversion disorders (F44)	223 (0.3)	100 (0.4)	323 (0.4)
Somatoform disorders (F45)	2900 (4.3)	1784 (7.5)	4684 (5.1)
Personality disorders (F60, F61)	746 (1.1)	524 (2.2)	1270 (1.4)
Enuresis/encopresis (F98.0, F98.1)	3538 (5.3)	958 (4.0)	4496 (4.9)
Stuttering/cluttering (F98.5, F98.6)	643 (1.0)	115 (0.5)	758 (0.8)
Sleeping disorders (F51, G47)	2377 (3.5)	1123 (4.7)	3500 (3.8)
Number of psychiatric comorbidities^a^, n (%)			
0	16,651 (24.8)	6282 (26.3)	22,933 (25.2)
1	19,307 (28.7)	6893 (28.8)	26,200 (28.8)
2	14,688 (21.9)	5012 (20.9)	19,700 (21.6)
≥ 3	16,548 (24.6)	5737 (24.0)	22,285 (24.5)

^a^Exclusively related to the 22 above-mentioned comorbidities

### Statistics

ADHD prevalence was calculated by dividing the number of individuals who met the criteria for ADHD case definition in each calendar year by the total number of eligible subjects (see previous section on study population). In addition to crude 1-year prevalence rates, standardized prevalence rates by age and sex were calculated based on the German population from December 2017 as reference. ADHD prevalences and proportions of interventions were also calculated stratified by sex. In age-stratified analyses, individuals were divided into the following age groups, which are associated with important developmental milestones: 3–6 years (preschool), 7–9 years (elementary school), 10–12 years (transition to high school), and 13–17 years (puberty). The proportions (%) of ADHD subjects with interventions or specific characteristics (e.g., comorbidities) were calculated by dividing the number of subjects who met each criterion by the number of all subjects who met ADHD criteria in the respective calendar year. Similar to ADHD prevalences, proportions of interventions were standardized by age and sex. Here, the reference population consisted of all children and adolescents in this study who were diagnosed with ADHD in 2017. Summary statistics comprised counts, percentages, means, and quartiles, as appropriate. All statistical analyses were conducted using SAS statistical software version 9.4.

## Results

### Characteristics of the study population

In 2017, a total of 2,156,733 children/adolescents (girls: 48.6%) were identified, who met the eligibility criteria. Hereof, 91,118 fulfilled the criteria for ADHD, resulting in a crude ADHD prevalence of 42.2/1000 (boys: 60.6; girls: 22.8, age- and sex-standardized: 42.8). The median age of children/adolescents with ADHD was 11 years (Q1: 9; Q3: 14). The proportion of patients within the age groups was 8.2% (3–6 years), 24.5% (7–9 years), 28.7% (10–12 years), and 38.6% (13–17 years). Across all data years, age- and sex-standardized prevalence of ADHD ranged between 37.2/1000 (2009) and 43.5/1000 (2015).

Table 1 shows the clinical characteristics of children/adolescents with ADHD in 2017. Overall, 74.8% of all children/adolescents with ADHD had at least one psychiatric comorbidity in 2017, 46.1% had two or more. The most common psychiatric comorbidities were conduct disorders (28.3%), specific developmental disorders of speech and language (21.9%) and of scholastic skills (19.7%). The proportion of comorbid children with ADHD dropped from 82.8% in those aged 3–6 years to 69.5% in those aged 13–17 years (data not shown).

### Treatment status of the study population

Table 2 depicts the treatment status of children/adolescents with ADHD in 2017. Overall, 50.2% received neither pharmacotherapy nor psychotherapy and 36.5% were treated with pharmacotherapy only. Psychotherapy only was documented for 6.8% and psychotherapy in combination with pharmacotherapy (multimodal treatment) for 6.5% of the children/adolescents with ADHD. Among those treated with psychotherapy (n = 12,116), 91.2% (n = 11,046) received guideline-based psychotherapy only, 6.3% (n = 770) received other non-drug psychiatric/psychotherapeutic treatment only and 2.5% (n = 300) received both (data not shown).

**Table 2:** Treatment status of children/adolescents with ADHD overall and by psychiatric comorbidities in 2017

	Proportion of patients with the respective treatment modality (%)				
	Number of patients	No treatment	Pharmacotherapy only	Psychotherapy only	Multimodal treatment
Overall	91,118	50.2	36.5	6.8	6.5
Sex					
Male	67,194	47.7	39.6	6.1	6.6
Female	23,924	57.2	28.0	8.9	5.9
Age group in years					
3–6	7498	89.1	4.6	5.2	1.1
7–9	22,298	61.0	25.6	7.6	5.7
10–12	26,139	44.7	39.9	7.6	7.8
13–17	35,183	39.0	47.7	6.2	7.1
Selected psychiatric comorbidities					
Depressive disorders (F32, F33, F41.2, F43.2)	11,651	36.2	31.9	18.0	14.0
Tic disorders (F95)	3030	41.2	40.6	8.0	10.2
Pervasive developmental disorders (F84.0, F84.1, F84.5, F84.8, F84.9)	5107	42.3	43.6	5.9	8.3
Conduct disorders (F90.1, F91, F92)	25,804	34.3	44.3	9.3	12.2
Phobic anxiety disorders (F40, F41.0, F41.1, F41.3, F41.8, F41.9, F93)	17,194	39.3	33.3	15.1	12.3
Number of psychiatric comorbidities^a^					
0	22,933	56.8	38.4	2.5	2.2
1	26,200	52.1	36.7	5.9	5.3
2	19,700	48.3	35.8	8.1	7.8
≥ 3	22,285	42.7	35.0	11.2	11.1

Percentages are row percentages

^a^Exclusively related to the 22 comorbidities mentioned in Table 1

The proportion of untreated children was higher in girls than in boys. The latter were more often treated with pharmacotherapy only, while girls more often received psychotherapy only. Across age groups, the proportion of untreated children dropped from 89.1% among those aged 3–6 years to 39.0% among those in the age group 13–17 years. Concurrently, the proportion of children with non-multimodal pharmacotherapy increased from 4.6% to 47.7%.

Regarding the psychiatric comorbidity status, the proportions of untreated or solely pharmacologically treated patients decreased with increasing numbers of further psychiatric diagnoses. Concurrently, the proportion of patients with psychotherapy only or with multimodal treatment more than quadrupled from those with no further diagnosis (2.5% and 2.2%) to those with more than two additional psychiatric diagnoses (11.2% and 11.1%).

**Fig. 1:**
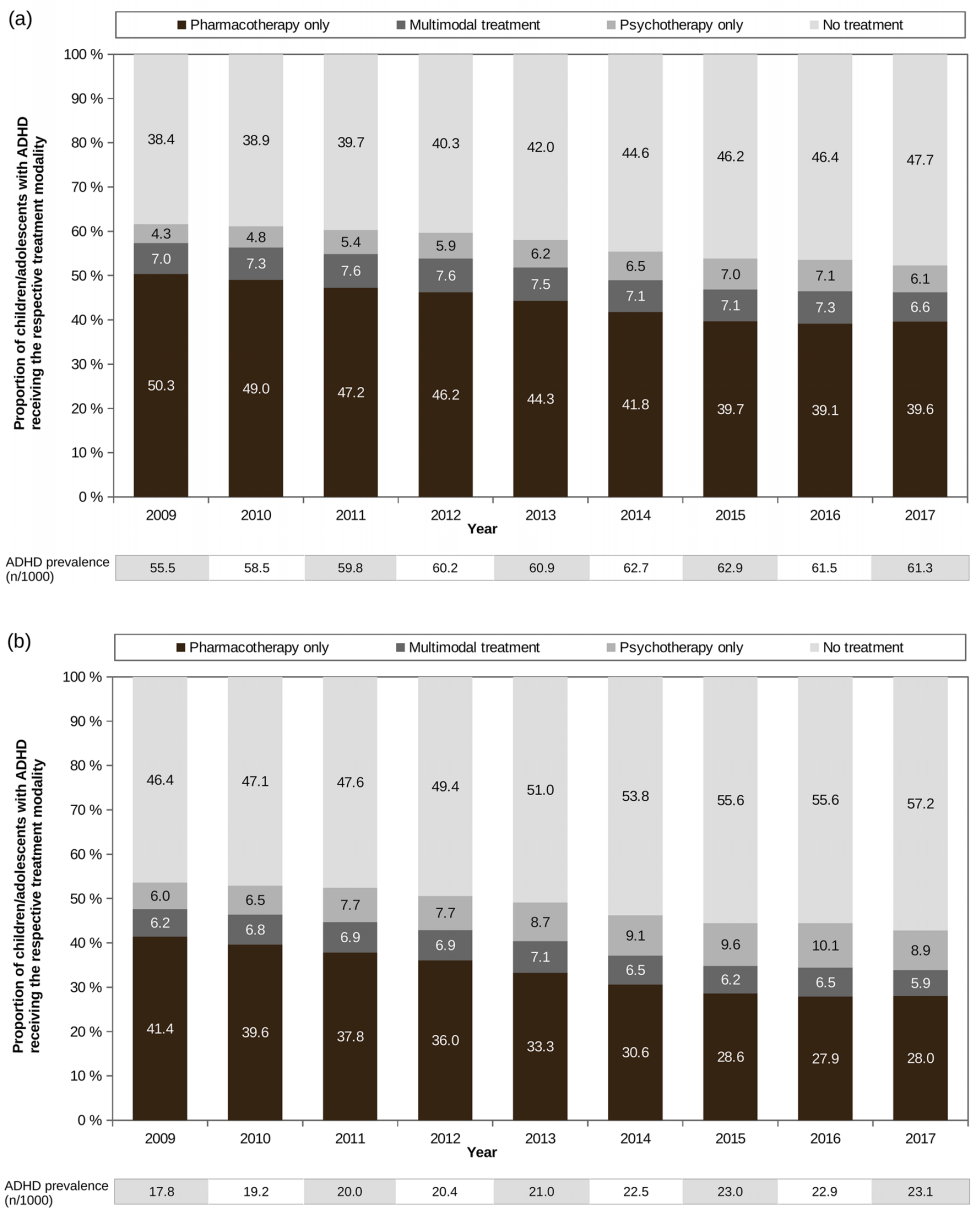
Age standardized proportions of treatment status of boys (**a**) and girls (**b**) with ADHD

Overall, from 2009 to 2017, the proportion of patients with only pharmacotherapy decreased from 48% to 36.5%, and the proportion of patients with no treatment concurrently increased from 40.5% to 50.2%. The fraction of patients with multimodal treatment ranged between 6.5% (2017) and 7.4% (2013), and the fraction of patients with only psychotherapy ranged between 4.8% (2009) and 7.9% (2016). These patterns were also seen in analyses stratified by sex (see Fig. 1).

## Discussion

We investigated the extent of (non-)multimodal treatment of children and adolescents with ADHD, based upon a large sample in German claims data.

### Proportion of children/adolescents receiving (non-)multimodal treatment

For the year 2017, almost half of all boys and more than 57% of all girls with ADHD were untreated, i.e., without pharmacotherapy or psychotherapy. For both sexes, these levels were reached after a steady increase of untreated patients from 2009 to 2017, corresponding to an increase of untreated patients by 26% (boys) and 24% (girls). This is consistent with the results showing that the majority of treated patients received pharmacotherapy only and that this fraction decreased concurrently from 2009 to 2014 (more pronounced in girls) before reaching a plateau in the years 2015–2017. Our findings therefore corroborate those of Akmatov et al. [13] whose analyses showed such a plateau already emerging for 2015 and 2016. This trend can probably be explained by the modification of the prescription rules for stimulants (such as methylphenidate) by the Federal Joint Committee (*Gemeinsamer Bundesausschuss*) in 2010. Since then, prescriptions of stimulants must not be based on ADHD symptoms alone but rather require a complete and comprehensive review of the patient’s medical history. Only specialized physicians are authorized to prescribe stimulants and to closely supervise general practitioners who are allowed to issue prescriptions in exceptional cases.

Irrespective of concomitant pharmacotherapy and also with consideration of other non-drug psychiatric/psychotherapeutic treatment our data show that with less than 14%, psychotherapy was only conducted in a fraction of children/adolescents with ADHD, with highest proportions of treated children among those aged 10–12 years. Higher treatment rates could have been expected since ADHD not only responds to psychosocial interventions but their benefit within the treatment regimen has been repeatedly confirmed [14–16] and it has been suggested that they might also lead to the reduction of the drug doses of pharmacotherapy [17].

It remains unclear why psychotherapy appears to be a neglected treatment option in our study population. One reason could be that in the short term, non-multimodal pharmacotherapy is similarly effective as multimodal treatment, which becomes more effective in the long run [14]. In general, patients with mental illness have been found to receive disorder-specific psychotherapy less often than could be expected [18], although significant socioeconomic benefits of psychotherapy in terms of cost reduction have been reported [19]. For ADHD, Haege et al. [20] recently pointed out that pharmacotherapy should be the first-line intervention in severe cases whereas in milder cases or preschool children, psychosocial interventions, including psychotherapy, should be preferred. In our data, this was not corroborated by the results on either guideline-based psychotherapy or other non-drug psychiatric/psychotherapeutic treatment among younger patients. Although side effects cannot be completely ruled out even with psychotherapy [21], reasons for the lack of use may be found elsewhere. For example, it is possible that alternative therapy options used for mild to moderate cases are not represented in our database. This might apply, for example, to occupational therapy, which is not captured in GePaRD and is frequently prescribed to ADHD patients in Germany despite lacking evidence of effectiveness [22]. Also, increased use of nutritional supplements in children with ADHD as recently reported [23] may be a reason why other evidence-based therapies are not used or delayed at the parents’ initiative. In this context, it must also be noted that the “psychotherapeutic culture” in Germany cannot be compared with that of other countries, such as the USA, where this form of therapy is traditionally more accepted. Similar effects have been recently demonstrated for South Korea where attitudes toward psychotherapy are more favorable compared to Germany [24]. Notably, also based on health claims data from the US, Gellad et al. [25] reported psychotherapy in one out of four medicated children with ADHD. Thus, considering potential stigmatization and comparatively higher therapy efforts, German parents might possibly give preference to a purely medicinal treatment more often. Also, though not specifically for children with ADHD, lack of information on treatment options has been identified as a potential treatment barrier [26]. In addition, and again not specific to ADHD, increased waiting times for a therapy slot for psychotherapy (in Germany: between four and five months on average) may also have contributed to a preference for solely medication-based over multimodal treatment.

In connection with previously mentioned findings, multimodal treatment was rather the exception than the rule in our study population, despite recommendations to the contrary [16, 27]. For more than eight out of ten patients with pharmacological therapy, no further interventions were coded. Conversely, half of all patients with psychotherapy received psychotherapy alone. The lack of multimodal treatment in our sample merits explanation, although some of the reasons for the underutilization of psychotherapy given above are likely to apply to multimodal treatment as well. It should also be mentioned that despite recommendations for the use of psychosocial interventions [28] data on their effectiveness cannot be regarded as consensual. Two recently published studies could not confirm a significant effect for psychosocial interventions, including psychotherapy. Lam et al. [29] reported data from a long-term observer-masked randomized controlled trial (RCT), comparing the effectiveness of pharmacotherapy in combination with cognitive behavioral group therapy (CBT) or standard clinical management. While the results indicated that pharmacotherapy was clearly superior to placebo treatment, CBT did not prove superior to standard clinical management. Similarly, Corbisiero et al. [30] presented findings from an RCT over 3 months, showing that during the observation time CBT did not outperform standard clinical management. It must be noted, however, that these studies were primarily directed at adult patients with ADHD. The data situation with regard to children therefore still needs clarification.

### Association of (non-)multimodal treatment with psychiatric comorbidities

Our data also clearly indicate that multimodal treatment—despite low absolute numbers—is more common in the presence of psychiatric multimorbidity. The proportion of patients who were treated with pharmacotherapy only was 8.8% lower (38.4% vs. 35.0%) in those with three or more psychiatric comorbidities than in those with ADHD alone. In contrast, the proportion of patients who were treated with psychotherapy was 4.5-fold higher (2.5% vs. 11.2%) across these groups and the proportion of patients with multimodal treatment was even more than fivefold higher (2.2% vs. 11.1%) correspondingly. Thus, these figures corroborate previous findings based on claims data which identified comorbid depression, and neurotic and somatoform, conduct, and emotional disorders as predictors for receiving both treatments instead of pharmacotherapy only in children newly diagnosed with ADHD [10]. However, it must be noted as an important limitation of our results that we did not assign therapeutic interventions to specific psychiatric (comorbid) diagnoses in our analyses. Such an assignment is not possible for multimorbid psychiatric patients due to the nature of health claims data. Therefore, our results only describe how many and which patients received multimodal therapy, but without being able to provide information on whether multimodal treatment was administered for a single psychiatric condition. This aspect should consequently be further investigated in future field studies.

### Increase of the proportion of multimodal treatment by the additional consideration of other non-drug psychiatric/psychotherapeutic treatment

Contrary to our expectations, the fraction of patients treated with other non-drug psychiatric/psychotherapeutic treatment was rather small. Thus, our data could not confirm other non-drug psychiatric/psychotherapeutic treatment as a low-threshold alternative or complementary therapy to the more regulated guideline-based psychotherapy. This outcome draws attention to a specific limitation of our study, which defined other non-drug psychiatric/psychotherapeutic treatment based on the algorithm proposed by Herpertz el al. [12]. It is possible that this algorithm—which to our knowledge has not been tested in routine care data so far—is too restrictive and that psychotherapy manifests itself already with a smaller number of coded measures. This is corroborated by recently published data on outpatient psychological therapies in children and adolescents [31]. In that study, interventions other than guideline-based psychotherapy accounted for a large proportion of psychotherapeutic treatment among the study population if the mere occurrence of corresponding billing codes was counted. Further studies and analyses are needed to investigate the impact of lower thresholds on the actual treatment rates.

Further limitations have to be kept in mind when interpreting our data. In addition to patient-centered interventions that are not captured in our database (e.g., occupational therapy), other psychosocial interventions are generally not billed for children in claims data (e.g., school interventions) either. Therefore, the proportion of patients receiving multimodal treatment might be underestimated in our study. Furthermore, according to our definition, multimodal treatment was already present if both forms of therapy took place within the same calendar year. Therefore, it would be theoretically conceivable that the times of drug and psychotherapeutic treatment did not necessarily overlap in some patients with multimodal treatment. However, we believe that possible bias effects here are rather small, since both pharmacotherapy and psychotherapy tend to follow a medium- to long-term therapeutic concept. Finally, as we have used a cross-sectional design, we were not able to factor therapies conducted prior to the respective data year. The consideration of treatment histories would have required a different study design which deliberately was not implemented in this study. For the same reason, this bears the risk that some patients with less than 6 months of observation in a given year did not have the chance to fulfill the psychotherapy algorithms, since billed therapy services were not counted beyond the respective year. However, we regard this risk as negligible, since in Germany the median duration of psychotherapy for children and adolescents is four quarters (unpublished data). Given the number of years we analyzed, it can therefore be expected that the values average out over the years, i.e., patients who do not (yet) meet the criteria due to a too short observation period in one year are very likely to be counted in the following year. Thus, there are further longitudinal studies needed to gain more insight into these aspects.

## Conclusions

The majority of children/adolescents with ADHD in Germany are treated either with pharmacotherapy alone or in combination with non-drug psychiatric/psychotherapeutic treatment, with a clear preponderance of non-multimodal care (pharmacotherapy) and a steady increase of untreated patients from 2009 to 2017. The concurrent decline in phamacologically treated patients was probably largely motivated by a restrictive modification of prescription guidelines. The proportions of patients with multimodal treatment substantially increased with increasing psychiatric multimorbidity. The consideration of other non-drug psychiatric/psychotherapeutic treatment in addition to guideline-based psychotherapy did not markedly increase the proportion of patients with psychotherapeutic interventions.

## Supplementary Information

**Additional file 1:** **Table S1.** Codes used for the identification of guideline-based psychotherapy.

**Additional file 2:** **Table S2.** Codes used for the identification of other non-drug psychiatric/psychotherapeutic treatments.

## Data Availability

Not applicable. As we are not the owners of the data we are not legally entitled to grant access to the data of the German Pharmacoepidemiological Research Database. In accordance with German data protection regulations, access to the data is granted only to BIPS employees on the BIPS premises and in the context of approved research projects. Third parties may only access the data in cooperation with BIPS and after signing an agreement for guest researchers at BIPS.

## Abbreviations

ADHD: Attention-deficit hyperactivity disorder
ATC: Anatomical-therapeutic-chemical
CBT: Cognitive behavioral group therapy
EBM: Einheitlicher Bewertungsmaßstab
GePaRD: German Pharmacoepidemiological Research Database
ICD-10-GM: International Statistical Classification of Diseases and Related Health Problems 10th Revision, German Modification
RCT: Randomized controlled trial
SHI: Statutory health insurance
