# Supplementary material for: Prevalence of multimodal treatment in children and adolescents with ADHD in Germany: a nationwide study based on health insurance data
Source: Child Adolesc Psychiatry Ment Health. 2021 Dec 18;15:76. doi: 10.1186/s13034-021-00431-0 (PMC8684641; doi:10.1186/s13034-021-00431-0)
Supplement: Supplementary file 1 — Additional file 1: Table S1. Codes used for the identification of guideline-based psychotherapy. [file 13034_2021_431_MOESM1_ESM.docx]

**Suppl. Table S1:** Codes used for the identification of guideline-based psychotherapy.

| **Code**^a^ | **Type of intervention** |
| --- | --- |
| 35111 | Relaxation techniques (single patient) |
| 35113 | Relaxation techniques (group treatment) |
| 35150 | Probationary session |
| 35200 | Depth psychotherapy (short-term, single patient) |
| 35201 | Depth psychotherapy (long-term, single patient) |
| 35202 | Depth psychotherapy (short-term, big group) |
| 35203 | Depth psychotherapy (long-term, big group) |
| 35205 | Depth psychotherapy (short-term, small group) |
| 35208 | Depth psychotherapy (long-term, small group) |
| 35401 | Depth psychotherapy (short-term 1, single patient) |
| 35402 | Depth psychotherapy (short-term 2, single patient) |
| 35405 | Depth psychotherapy (long-term, single patient) |
| 35503 | Depth psychotherapy (short-term, group, 3 participants) |
| 35504 | Depth psychotherapy (short-term, group, 4 participants) |
| 35505 | Depth psychotherapy (short-term, group, 5 participants) |
| 35506 | Depth psychotherapy (short-term, group, 6 participants) |
| 35507 | Depth psychotherapy (short-term, group, 7 participants) |
| 35508 | Depth psychotherapy (short-term, group, 8 participants) |
| 35509 | Depth psychotherapy (short-term, group, 9 participants) |
| 35513 | Depth psychotherapy (long-term, group – 3 participants) |
| 35514 | Depth psychotherapy (long-term, group – 4 participants) |
| 35515 | Depth psychotherapy (long-term, group – 5 participants) |
| 35516 | Depth psychotherapy (long-term, group – 6 participants) |
| 35517 | Depth psychotherapy (long-term, group – 7 participants) |
| 35518 | Depth psychotherapy (long-term, group – 8 participants) |
| 35519 | Depth psychotherapy (long-term, group – 9 participants) |
| 35210 | Analytical psychotherapy (single patient) |
| 35211 | Analytical psychotherapy (group treatment) |
| 35411 | Analytical psychotherapy (short-term 1, single patient) |
| 35412 | Analytical psychotherapy (short-term 2, single patient) |
| 35415 | Analytical psychotherapy (long-term, single patient) |
| 35523 | Analytical psychotherapy (short-term, group, 3 participants) |
| 35524 | Analytical psychotherapy (short-term, group, 4 participants) |
| 35525 | Analytical psychotherapy (short-term, group, 5 participants) |
| 35526 | Analytical psychotherapy (short-term, group, 6 participants) |
| 35527 | Analytical psychotherapy (short-term, group, 7 participants) |
| 35528 | Analytical psychotherapy (short-term, group, 8 participants) |
| 35529 | Analytical psychotherapy (short-term, group, 9 participants) |
| 35533 | Analytical psychotherapy (long-term, group, 3 participants) |
| 35534 | Analytical psychotherapy (long-term, group, 4 participants) |
| 35535 | Analytical psychotherapy (long-term, group, 5 participants) |
| 35536 | Analytical psychotherapy (long-term, group, 6 participants) |
| 35537 | Analytical psychotherapy (long-term, group, 7 participants) |
| 35538 | Analytical psychotherapy (long-term, group, 8 participants) |
| 35539 | Analytical psychotherapy (long-term, group, 9 participants) |
| 35220 | Cognitive behavioral therapy (short-term, single patient) |
| 35221 | Cognitive behavioral therapy (long-term, single patient) |
| 35222 | Cognitive behavioral therapy (short-term, small group) |
| 35223 | Cognitive behavioral therapy (long-term, small group) |
| 35224 | Cognitive behavioral therapy (short-term, big group) |
| 35225 | Cognitive behavioral therapy (long-term, big group) |
| 35421 | Cognitive behavioral therapy (short-term 1, single patient) |
| 35422 | Cognitive behavioral therapy (short-term 2, single patient) |
| 35425 | Cognitive behavioral therapy (long-term, single patient) |
| 35543 | Cognitive behavioral therapy (short-term, group, 3 participants) |
| 35544 | Cognitive behavioral therapy (short-term, group, 4 participants) |
| 35545 | Cognitive behavioral therapy (short-term, group, 5 participants) |
| 35546 | Cognitive behavioral therapy (short-term, group, 6 participants) |
| 35547 | Cognitive behavioral therapy (short-term, group, 7 participants) |
| 35548 | Cognitive behavioral therapy (short-term, group, 8 participants) |
| 35549 | Cognitive behavioral therapy (short-term, group, 9 participants) |
| 35553 | Cognitive behavioral therapy (long-term, 3 participants) |
| 35554 | Cognitive behavioral therapy (long-term, 4 participants) |
| 35555 | Cognitive behavioral therapy (long-term, 5 participants) |
| 35556 | Cognitive behavioral therapy (long-term, 6 participants) |
| 35557 | Cognitive behavioral therapy (long-term, 7 participants) |
| 35558 | Cognitive behavioral therapy (long-term, 8 participants) |
| 35559 | Cognitive behavioral therapy (long-term, 9 participants) |

^a^ Outpatient treatment / diagnostic procedures are coded using claim codes for outpatient services and procedures [Einheitlicher Bewertungsmaßstab, EBM]
