# Supplementary material for: Prevalence of multimodal treatment in children and adolescents with ADHD in Germany: a nationwide study based on health insurance data
Source: Child Adolesc Psychiatry Ment Health. 2021 Dec 18;15:76. doi: 10.1186/s13034-021-00431-0 (PMC8684641; doi:10.1186/s13034-021-00431-0)
Supplement: Supplementary file 2 — Additional file 2: Table S2. Codes used for the identification of other non-drug psychiatric/psychotherapeutic treatments. [file 13034_2021_431_MOESM2_ESM.docx]

**Suppl. Table S2:** Codes used for the identification of other non-drug psychiatric/psychotherapeutic treatments.

| **Code**^a^ | **Type of intervention** |  | **Category**^b^ |
| --- | --- | --- | --- |
| 14220 | Medical consultation, advisory, diagnostic clarification (single patient) |  | 2 |
| 14221 | Group treatment |  | 1 |
| 14310 | Functional development therapy (single patient) |  | 2 |
| 14311 | Functional development therapy (group treatment) |  | 2 |
| 21220 | Medical consultation, advisory, diagnostic clarification (single patient) |  | 2 |
| 21221 | Psychiatric treatment (group treatment) |  | 1 |
| 22220 | Psychotherapeutic interview |  | 2 |
| 23220 | Psychotherapeutic interview |  | 2 |

^a^ Outpatient treatment / diagnostic procedures are coded using claim codes for outpatient services and procedures [Einheitlicher Bewertungsmaßstab, EBM]

^b^ 1=Duration at least 20 minutes per unit, 2=duration less than 20 minutes per unit. Psychotherapeutic care was assumed if a) at least six identical codes of category 1 within 183 days, or b) at least twelve identical codes of category 2 within 183 days and at least two identical codes per day were billed
